# RETRACTION: Expression Profiling of Abiotic Stress‐Inducible Genes in response to Multiple Stresses in Rice (Oryza sativa L.) Varieties with Contrasting Level of Stress Tolerance

**DOI:** 10.1155/bmri/9851735

**Published:** 2026-07-13

**Authors:** BioMed Research International

RETRACTION: S. Basu and A. Roychoudhury, “Expression Profiling of Abiotic Stress‐Inducible Genes in response to Multiple Stresses in Rice (Oryza sativa L.) Varieties with Contrasting Level of Stress Tolerance”, *BioMed Research International*, vol. 2014 (2014). 10.1155/2014/706890.

The above article, published online on 7 July 2014 in Wiley Online Library (https://wileyonlinelibrary.com/), has been retracted following the agreement of the journal’s Chief Editor Yoel A. Klug; and John Wiley & Sons Ltd.

The retraction has been agreed following an investigation of the concerns raised by a third party [1]. The investigation identified concerns related to the RT‐PCR data shown in the article as follows:

Figure 2a: The bands representing the expression HKT‐1 in the Pokkali group after 16 and 24 hours appear identical to the bands showing the expression of HKT‐1 in the Pokkali group after 6 and 24 hours in Figure 3a.

Figure 4a: The bands denoting the expression of Rab16a are unexpectedly similar to the bands corresponding to SOS‐3 and Rab16a in Figure 5a, which were obtained from a different sample.

Further investigation undertaken by the publisher has identified additional instances of overlap which are summarized below:

Figure 2a: The bands showing the expression of SOS‐3 and NAC‐1 in the Pokkali group appear identical to the SOS‐3 and Rab‐16a bands for the IR‐29 and Pokkali groups in Figure 3b.

Figure 3a: The bands denoting the expression of CRT/DREBP and Rab16a are similar to the NAC‐1 and SAPK7 bands in Figure 5b.

As a result of the investigation, the data and conclusions of this article are considered unreliable. Therefore, the article is retracted.

The authors were informed of the retraction but did not provide a response.
